# Theaflavins as Electrolyte Additives for Inhibiting Zinc Dendrites and Hydrogen Evolution in Aqueous Zinc-Ion Batteries

**DOI:** 10.3390/ijms26199399

**Published:** 2025-09-26

**Authors:** Xiao Zhang, Ting Cheng, Chen Chen, Fuqiang Liu, Fei Wu, Li Song, Baoxuan Hou, Yuan Tian, Xin Zhao, Safi Ullah, Rui Li

**Affiliations:** 1 School of Environmental Ecology, The City Vocational College of Jiangsu, Jiangsu Engineering and Technology Centre for Ecological and Environmental Protection in Urban and Rural Water Environment Management and Low Carbon Development, Nanjing 210017, China; zhangxiao7376@sina.com (X.Z.); wnchengting@sina.com (T.C.); nlsongli@163.com (L.S.); 2 State Key Laboratory of Pollution Control and Resource Reuse, School of Environment, Nanjing University, Nanjing 210023, China; lfq@nju.edu.cn; 3 School of Environmental and Chemical Engineering, Jiangsu University of Science and Technology, Zhenjiang 212100, China; wufei1224wf@hotmail.com (F.W.); hbx.1999@outlook.com (B.H.); ttyy1974.ok@163.com (Y.T.); imsafi222@gmail.com (S.U.); leerui0314@outlook.com (R.L.); 4 Key Laboratory of Agro-Forestry Environmental Processes and Ecological Regulation of Hainan Province, School of Environmental Science and Engineering, Hainan University, Haikou 570228, China

**Keywords:** aqueous battery, zinc ion, theaflavins, tea, electrolyte additives

## Abstract

The cycling stability and widespread practical implementation of aqueous zinc ion batteries (AZIBs) are impeded by dendrite growth and the hydrogen evolution reaction (HER). Herein, theaflavins, a low-cost organic bio-compounds and a major component of tea, were innovatively introduced as an electrolyte additive for AZIBs to address these challenges. When added into the electrolyte, theaflavins, with their strong de-solvation capability, facilitated the more uniform and stable diffusion of zinc ions, effectively suppressing dendrite formation and HER. This, in turn, significantly enhanced the coulombic efficiency (>95% in Zn/Cu system) and the stability of the zinc deposition/stripping process in Zn/Zn system. The Zn/Zn symmetric battery system stably cycled for approximately 3000 h at current densities of 1 mA/cm^2^. Compared with H_2_O molecules, theaflavins exhibited a narrower LUMO and HOMO gap and higher adsorption energy on zinc surfaces. These properties enabled theaflavins to be preferentially adsorbed onto zinc anode surfaces, forming a protective layer that minimized direct contact between water molecules and the zinc surface. This layer also promoted the electron transfer associated with zinc ions, thereby greatly enhancing interfacial stability and significantly mitigating HER. When 10 mmol/L of theaflavins was present in the electrolyte, the system exhibited lower impedance activation energy, a smoother zinc ion deposition process, reduced corrosion current, and higher HER overpotential. Furthermore, incorporating theaflavins into the electrolyte enhanced the vanadium redox reaction and accelerated zinc ion diffusion, thereby significantly improving battery performance. This work explores the design of a cost-effective electrolyte additive, providing essential insights for the progress of practical AZIBs.

## 1. Introduction

In the realm of renewable energy technologies [1,2], battery energy storage emerges as a crucial role, where new energy vehicles have ascended to prominence as a key application [3,4]. Currently, lithium-ion batteries (LIBs) are the predominant power source and are also widely utilized in portable electronic devices [5]. However, the limitations of these technologies are becoming increasingly apparent [6,7]. In particular, the rapid depletion of lithium resources poses a significant challenge. Additionally, safety concerns such as thermal runaway, which can trigger explosions and fires, are raising alarm bells in the industry [8,9]. Consequently, the development of safer, more reliable, and high-capacity alternatives to LIBs has emerged as a critical research focus in the field of sustainable energy storage. Aqueous zinc-ion batteries (AZIBs) have recently emerged as a promising next-generation battery system [10,11], offering a theoretical capacity of up to 820 mAh·g^−1^ [12,13]. Compared to conventional LIBs, AZIBs exhibit two major advantages [14,15]: (a) the use of an aqueous electrolyte significantly enhances safety by reducing the risk of thermal runaway [16,17], and (b) zinc is more abundantly available in the Earth’s crust and is considerably more cost-effective than lithium [16,17]. These advantages have driven extensive research on AZIBs, demonstrating their excellent theoretical performance and promising application potential in energy storage.

Nevertheless, the cycling stability and widespread practical implementation of AZIBs is impeded by two significant obstacles. The first is the formation of zinc dendrites, which can cause short-circuiting and severely impact the battery’s lifespan and safety [18]. In the charge–discharge process, dendrites can form due to the irregular deposition of zinc, which results from ion migration and the incompatibility of crystal growth. As these dendrites grow vertically in an uncontrolled manner, they may ultimately penetrate the separator, leading to internal short-circuits and subsequent battery failure [19]. The second limitation pertains to water electrolysis [20]. As the cycling time extends, it is highly likely to result in gas accumulation, which in turn can cause battery expansion. A significant amount of work has been conducted in recent years to tackle the aforementioned issues with AZIBs. These research efforts primarily involve zinc anode surface modification [21], structural design [22], separator modification [23], electrolyte optimization, etc. As the electrolyte is crucial for facilitating the connection between electrodes and ions in AZIBs, electrolyte optimization is regarded as a highly practical approach with significant application value. Currently, the incorporation of organic additives into the electrolyte has proven to be an exceptionally effective strategy for addressing these challenges [24]. The additive strategy is notable for being both simple and highly effective, and it provides the dual advantages of convenient preparation and a reasonable cost. These additives serve multiple functions, including promoting ion de-solvation, enhancing zinc ion and charge transport, and thereby improving the electrochemical activity of the battery. Additionally, they adhere to the zinc surface, forming a protective layer that effectively inhibits the growth of zinc dendrites and suppresses the hydrogen evolution reaction (HER), thereby enhancing battery stability and safety [25,26,27,28].

Relevant studies have demonstrated that organic compounds employed as additives should possess high water solubility and feature a wealth of hydrophilic functional groups [29,30]. This makes many bio-based natural organic compounds highly promising candidates for additive applications [31,32,33]. These organic molecules, when used as additives in AZIBs, have been demonstrated to effectively enhance electrochemical performance and significantly extend the lifetime of batteries. Despite considerable advancements in enhancing the lifespan of AZIBs using various additives, the development of high-efficiency, environmentally friendly electrolyte additives that can significantly limit the formation of zinc dendrites and synergistically regulate the electrode interface remains a highly challenging endeavor. Tea is one of the most widely consumed beverages globally and contains a variety of non-toxic organic compounds. If the organic compounds in tea could be harnessed to optimize the reaction processes of AZIBs, this would not only expand the application potential of tea-based solutions, but also enhance the stable operation of AZIBs, reduce the biological toxicity of their electrolytes, and ultimately advance the development of AZIBs technology. As a key polyphenol oxidation product in tea, theaflavins exhibit high water solubility and contain abundant hydrophilic hydroxyl groups, making them a promising candidate for use as an additive in AZIBs.

Herein, theaflavins, a major component of tea and an organic bio-compound additive, were innovatively introduced into the electrolyte of AZIBs to limit the formation of zinc dendrites and suppress the HER. Density functional theory (DFT) calculations were initially performed to determine the electrostatic potential, the adsorption energy, and the highest occupied molecular orbital (HOMO) and lowest unoccupied molecular orbital (LUMO) levels of both H_2_O and theaflavins molecules. A variety of characterization techniques and electrochemical experiments were employed to analyze the properties of the electrolyte solution, the surface characteristics of zinc sheets, the inhibitory effects on zinc dendrite formation and HER, and the stabilizing effects of theaflavins on zinc stripping and deposition cycles. Experiments and theoretical simulation confirmed that theaflavins served as an effective additive in AZIBs, significantly inhibiting zinc dendrite formation and HER, thereby enhancing battery stability and overall performance. The Zn/Zn symmetric system exhibited stable cycling for approximately 3000 h at a current density of 1 mA/cm^2^. This study offers a valuable strategy for enhancing the electrochemical performance and extending the lifespan of zinc electrodes in AZIBs.

## 2. Results and Discussion

**Figure 1:**
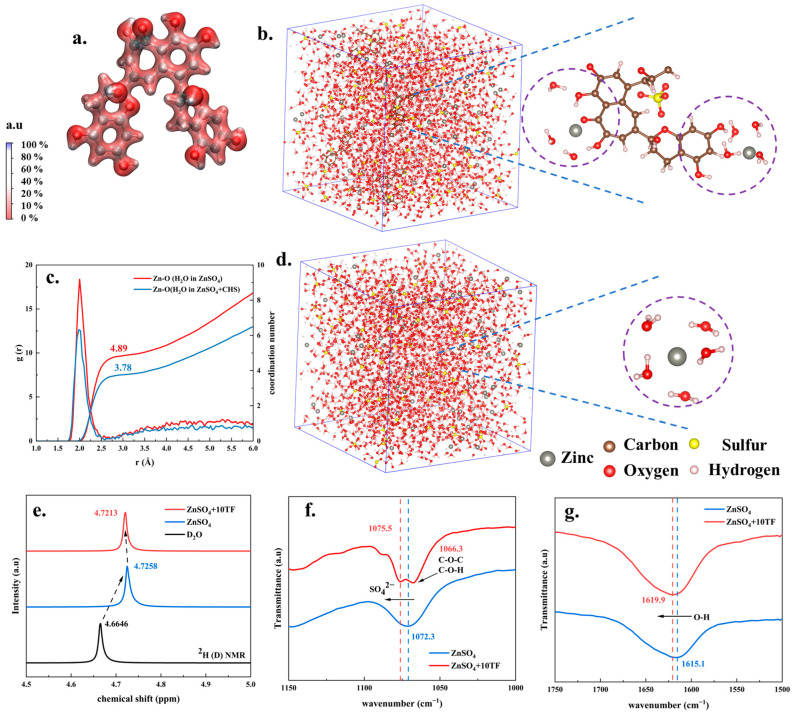
The electrostatic potential distribution of theaflavins molecules (**a**); The 3D snapshot of ZnSO_4_+10TF (**b**) and ZnSO_4_ (**d**) electrolyte and partial enlarged images representing Zn^2+^ solvation structure from MD (molecular dynamics) computational simulation; The RDF (radial distribution function) of O atom in H_2_O molecule as Zn^2+^ was center and calculated coordination number from MD computational simulation (**c**); ^2^H (D) NMR (nuclear magnetic resonance) analysis results of ZnSO_4_ and ZnSO_4_+10TF solutions (**e**); FT-IR (fourier transform infrared) analysis results detail of ZnSO_4_ and ZnSO_4_+10TF solutions (**f**): 1000–1150 cm^−1^ and (**g**): 1750–1500 cm^−1^).

Figure 1a presents the theoretical calculation results of the electrostatic potential distribution of the theaflavin molecule. As observed, the theaflavin molecule contained multiple hydroxyl groups, where hydrogen atoms exhibited a higher electrostatic potential (indicating a tendency to accept electrons), while oxygen atoms displayed a lower electrostatic potential (indicating a tendency to donate electrons). These hydroxyl groups served as abundant active sites for interactions between theaflavins, Zn^2+^, and H_2_O, significantly influencing the solvation effects of electrolytes [31,34]. Furthermore, the solvation effects of theaflavins on electrolytes were investigated through molecular dynamics simulations, NMR, and FT-IR spectroscopy. Figure 1b,d present the visualization results of the molecular dynamics calculations. In the ZnSO_4_ electrolyte system, five water molecules were observed within the solvation shell surrounding the zinc ion. In contrast, in the ZnSO_4_+TF electrolyte system, the hydrophilic groups of theaflavin molecules were able to penetrate the solvation shell of water molecules surrounding Zn^2+^, thereby effectively modifying the solvation structure of Zn^2+^ [35,36]. The radial distribution function (RDF) of the oxygen atom in the H_2_O molecule, centered around Zn^2+^, revealed the presence of a solvation shell of water molecules at approximately 2 Å. The integrated coordination number was about 4.89, which aligned with the visualized results presented in Figure 1c, and was consistent with findings reported in previous studies [37,38]. Upon the addition of the theaflavin molecule, the solvation shell remained centered at around 2 Å; however, the coordination number decreased to approximately 3.78. This reduction was likely attributable to the hydrophilic groups of theaflavins penetrating the Zn^2+^ solvation shell, displacing some water molecules [38,39]. Furthermore, as shown in Figure 1e, the ^2^H peak in D_2_O solvent was initially observed at a chemical shift of 4.6646 ppm. Upon the dissolution of ZnSO_4_ in the system, the ^2^H peak shifted to 4.7258 ppm. This phenomenon, also reported in related studies, was primarily attributed to the strong coordination between Zn^2+^ and D_2_O (H_2_O), which reduced the number of free water molecules [35]. The strong coordination between Zn^2+^ and D_2_O (H_2_O) was expected to decrease electron density and weaken proton shielding [38]. Subsequently, upon the addition of trace amounts of theaflavins (10 mmol/L), the ^2^H peak shifted slightly back to 4.7213 ppm. This shift suggested that theaflavins weakened the strong coordination between Zn^2+^ and D_2_O (H_2_O), allowing some water molecules to return to a free state [40]. Furthermore, FT-IR analysis also provided evidence of theaflavins’ influence on solvation.

As shown in Figure 1f, the FT-IR spectrum of the ZnSO_4_ solution exhibited a distinct absorption peak at 1072.3 cm^−1^, corresponding to the vibrational stretching of SO_4_^2−^. Upon the introduction of theaflavins into the system, additional absorption peaks attributed to C-O-H side groups emerged at approximately 1066.3 cm^−1^, confirming the presence of theaflavins. Simultaneously, the SO_4_^2−^ absorption peak shifted to 1075.5 cm^−1^, indicating a reduction in the constraint around SO_4_^2−^. This shift was likely due to the weakened electrostatic interaction between Zn^2+^ and SO_4_^2−^ induced by the presence of theaflavins [41]. Furthermore, as illustrated in Figure 1g, the absorption peak corresponding to the bending vibration of O-H in water, initially located at 1615.1 cm^−1^, shifted to a higher wavenumber (1619.9 cm^−1^) after the addition of theaflavins, suggesting an increase in the proportion of free water molecules in the system. This phenomenon was likely attributed to theaflavins occupying the active sites originally held by water molecules within the solvent structure, thereby increasing the amount of free water [32]. Based on the combined results of MD simulations, NMR, and FT-IR, it was evident that the addition of theaflavins disrupts the solvation structure between Zn^2+^ and SO_4_^2−^, H_2_O. This disruption facilitated the presence of more free Zn^2+^ and H_2_O molecules in the electrolyte, thereby enhancing Zn^2+^ ion diffusion within the system.

**Figure 2:**
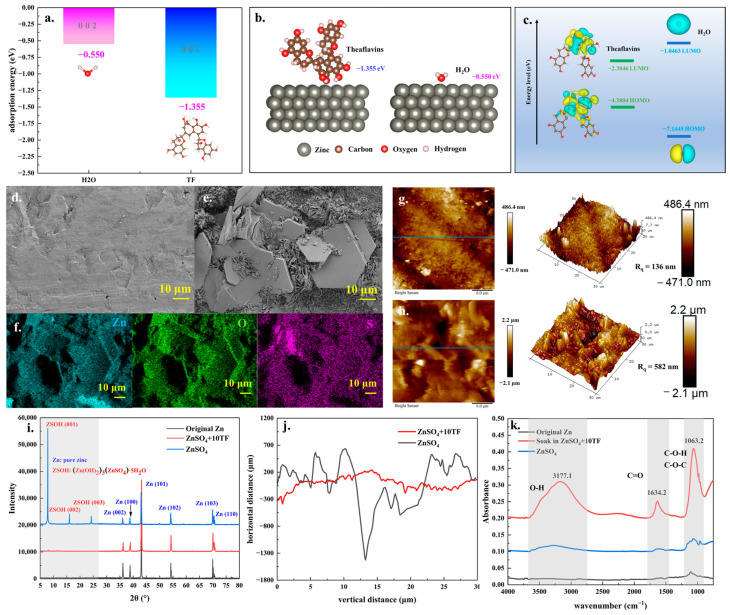
The theoretical adsorption energy DFT (density functional theory) calculation results (**a**) and optimal configurations (**b**) of H_2_O and theaflavins molecules on the 002 crystal surface of zinc sheet; the theoretical LUMO (highest occupied molecular orbital) and HOMO (lowest unoccupied molecular orbital) DFT calculation results of theaflavins and H_2_O molecules (**c**); the SEM (scanning electron microscope) analysis results of a zinc sheet soaked in ZnSO_4_ (**d**) and ZnSO_4_+10TF solutions (**e**); the EDX (energy dispersive X-ray)-mapping analysis results of Figure 2e’s area (**f**); the AFM (atomic force microscopy) analysis results of a zinc sheet soaked in ZnSO_4_ (**h**) and ZnSO_4_+10TF solutions (**g**); the XRD (X-ray diffraction) analysis results of a zinc sheet soaked in ZnSO_4_ (**g**) and ZnSO_4_+10TF solutions (**i**); the test results of roughness along the analysis line in Figure 2g,h (**j**); the FT-IR (fourier transform infrared) of ATR (attenuated total reflection) model analysis results of a zinc sheet surface soaked in ZnSO_4_ and ZnSO_4_+10TF solutions (**k**).

To gain deeper insight into the effect of theaflavins on the battery reaction process, DFT calculations were conducted to investigate the adsorption behavior of theaflavins and H_2_O molecules on the surface of zinc sheets. Figure 2a,b present the adsorption energies and optimized adsorption configurations of both species on the zinc 002 crystal surface. The results indicated that the theaflavins exhibited a stronger adsorption affinity toward the zinc 002 surface compared to H_2_O molecules. Similar trends were also observed on the zinc 100 and 101 surfaces, as shown in Figures S1 and S2. These findings clearly indicated that theaflavins were more favorably adsorbed onto the surface of zinc sheets than H_2_O molecules. In general, the preferential adsorption of organic molecules such as theaflavins on the surface of zinc sheets could inhibit the HER, which relied on the adsorption of H_2_O molecules, while simultaneously promoting the uniform deposition of zinc ions [42,43]. Theoretical calculations of the LUMO and HOMO energy levels for theaflavins and H_2_O molecules were presented in Figure 2c. Compared to H_2_O molecules, theaflavins displayed a lower LUMO energy level (−2.3846 eV) and a higher HOMO energy level (−4.3804 eV), facilitating electron transfer from the theaflavins molecules to zinc ions in the electrolyte [36,44]. Furthermore, the XRD analysis results of zinc sheets soaked in 2 mol/L ZnSO_4_ and 2 mol/L ZnSO_4_ + 10 mmol/L theaflavins solutions were presented in Figure 2i. After immersion in ZnSO_4_ solution, distinct diffraction peaks corresponding to (Zn(OH)_2_)_3_(ZnSO_4_)·5H_2_O) (PDF. 78-0246) were observed, indicating that corrosion reaction occurred on the surface of zinc sheet during this soaking process. In contrast, the XRD pattern of the zinc sheet soaked in the ZnSO_4_ solution containing theaflavins showed no additional diffraction peaks other than those associated with metallic zinc, suggesting effective inhibition of corrosion by theaflavins. This conclusion was further supported by SEM-EDX analysis. As shown in Figure 2e, the surface of the zinc sheet immersed in ZnSO_4_ solution exhibited abundant corrosion products, confirming significant surface degradation. Elemental distribution analysis using EDX mapping (Figure 2f) revealed that the primary constituents of the surface corrosion products were zinc, sulfur, and oxygen, which aligned well with the molecular formula of (Zn(OH)_2_)_3_(ZnSO_4_)·5H_2_O. In contrast, the zinc sheet soaked in the ZnSO_4_ solution containing theaflavins retained a relatively smooth surface morphology, with the dominant surface elements being zinc, carbon, and oxygen (Figure S3). The presence of carbon was attributed to the preferential adsorption of theaflavins on the zinc surface. To further evaluate the surface characteristics, atomic force microscopy (AFM) was employed to assess the surface roughness of the soaked zinc sheets, as shown in Figure 2g. Comparative analysis revealed that the surface roughness of the zinc sheet soaked in the ZnSO_4_+theaflavins solution (ranging from −471.0 to 486.4 nm, with a root mean square roughness R_q_ = 136 nm) was significantly lower than that of the sheet soaked in pure ZnSO_4_ solution (ranging from −2.1 to 2.2 μm, R_q_ = 582 nm). This observation was further supported by the AFM profiles shown in Figure 2j, where the vertical height fluctuations along the scan lines were markedly greater for the sheet soaked in ZnSO_4_ solution. These AFM results clearly demonstrated that the surface of the zinc sheet treated with theaflavins remained considerably smoother, indicating effective inhibition of corrosion and deposition of reaction products.

Furthermore, Figure 2k presented the ATR-IR spectral analysis of the pristine zinc sheet and those soaked in different electrolyte solutions. As shown, the spectra of both the original zinc sheet and the one soaked in ZnSO_4_ solution exhibited only a few weak organic absorption peaks, indicating the absence of significant organic species on their surfaces [30]. In contrast, the zinc sheet soaked in the ZnSO_4_+Theaflavins solution displayed prominent absorption bands at 3177.1, 1634.2, and 1063.2 cm^−1^, respectively. The strong peak at 3177.1 cm^−1^ could be attributed to O–H stretching vibrations associated with polyhydroxy groups [45], while the peak at 1634.2 cm^−1^ corresponded to C=O stretching vibrations within aromatic rings. Additionally, the absorption at 1063.2 cm^−1^ was ascribed to C–O–H bending vibrations from side groups [46,47]. These spectral features confirmed the successful adsorption of theaflavins on the zinc surface. Given that these organic functional groups were present in the theoretical molecular structure of theaflavins, and the same three characteristic absorption peaks were observed in the FT-IR spectrum of the pure theaflavins solution, it could be inferred that a substantial amount of theaflavin molecules was adsorbed onto the zinc sheet surface following immersion in the ZnSO_4_+Theaflavins solution. Combining both theoretical and experimental findings, it was evident that theaflavins presented a stronger adsorption affinity to the zinc surface than water molecules and are more capable of facilitating electron transfer. This preferential adsorption behavior effectively inhibited the corrosion of zinc sheets in ZnSO_4_ solution and preserved surface smoothness. These properties were highly advantageous for enhancing the stability and performance of zinc-ion batteries.

**Figure 3:**
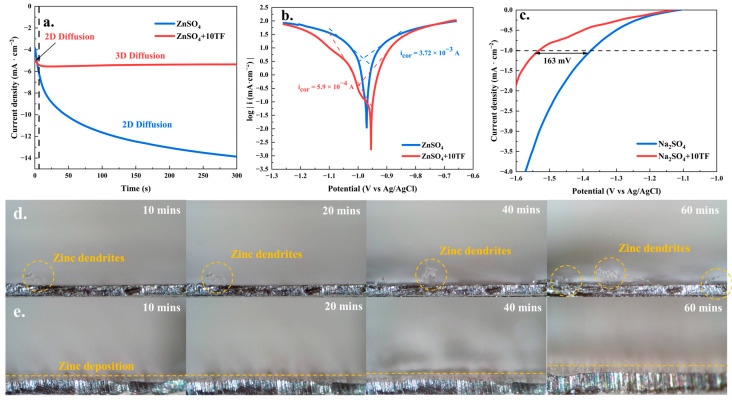
The chronoamperometry analysis results (**a**); the Tafel analysis results (**b**); the pure HER (hydrogen evolution reaction) analysis results in the Na_2_SO_4_ system (**c**) and the in situ optical microscopy of different deposition systems ((**d**) ZnSO_4_ and (**e**) ZnSO_4_+10TF).

The electrochemical performance results were presented in Figure 3. Further insights were obtained from the chronoamperometry (CA) measurements, as shown in Figure 3a. In the ZnSO_4_ system, the current continuously increased over the entire testing period (0–300 s), suggesting that zinc ions underwent an uncontrolled two-dimensional (2D) diffusion process. During this process, zinc ions preferentially migrated toward regions of higher electric field intensity, leading to the formation of non-uniform and irregular zinc deposits. This uncontrolled deposition behavior ultimately contributed to the nucleation and growth of zinc dendrites [48]. In contrast, within the ZnSO_4_+10TF system, the current rapidly reached a steady state (within approximately 11 s) and remained stable thereafter, indicating a controlled three-dimensional (3D) diffusion process. Such behavior typically resulted in smooth and uniform zinc deposition, thereby effectively suppressing dendrite formation. Additionally, Figure 3b presented the results of Tafel analysis and the corresponding corrosion current calculations. It was clear that the incorporation of theaflavins into the electrolyte significantly reduced the corrosion current from 3.72 mA to 0.59 mA, demonstrating a substantial enhancement in the corrosion resistance of the zinc electrode surface [40,49]. Finally, Figure 3c illustrated the HER curve in the Na_2_SO_4_ electrolyte system, which reflected the pure HER process without zinc deposition. As shown, the addition of theaflavins resulted in an increase in HER overpotential by 164 mV at −1 mA·cm^−2^, suggesting that theaflavins restricted the HER process. This inhibition was highly beneficial for the overall reaction stability of zinc-ion batteries. Furthermore, the impact of theaflavins on zinc deposition behavior was observed through in situ optical microscopy of different deposition systems (1 mA·cm^−2^; 1 mAh·cm^−2^). As seen in Figure 3d, irregular zinc dendrite formation was clearly visible in the ZnSO_4_ system, with dendrites continuing to grow throughout the reaction. In contrast, no irregular deposits were observed in the ZnSO_4_+10TF system (Figure 3e), indicating that zinc was deposited in a more controlled and uniform manner on the zinc sheet surface. Overall, the incorporation of theaflavins reduced impedance activation energy and corrosion current, optimized zinc ion diffusion behavior, inhibited the HER process, and suppressed dendrite formation, all of which contributed to the stable operation of zinc-ion batteries.

**Figure 4:**
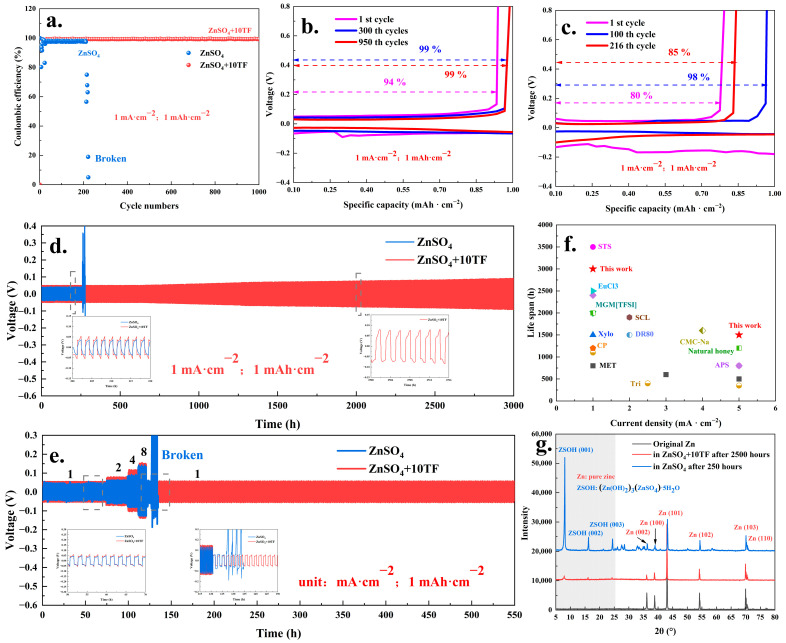
The Zn-Cu asymmetric battery system testing results ((**a**): 1 mA·cm^−2^ and 1 mAh·cm^−2^); The voltage profiles of Zn-Cu asymmetric battery system with ZnSO_4_+10TF (**b**) and ZnSO_4_ (**c**) systems; long Zn-Zn symmetric battery system testing results ((**d**): 1 mA·cm^−2^ and 1 mAh·cm^−2^); the rate performance of Zn-Zn symmetric battery system ((**e**): unit: mA·cm^−2^ and 1 mAh·cm^−2^); the comparison of stability efficiency with other battery additives (**f**) [38,39,40,48,49,50,51,52,53,54] and the XRD (X-ray diffraction) analysis results of Zn sheet after Zn-Zn symmetric battery system testing (**g**).

Based on the aforementioned results, the incorporation of theaflavin into the electrolyte effectively suppressed the formation of zinc dendrites and inhibited the HER. Subsequently, the electrochemical performance of the button battery system was investigated, as illustrated in Figure 4. Figure 4a–c present the test results of a Zn-Cu asymmetric battery system operated at a current density of 1 mA·cm^−2^ with a capacity of 1 mAh·cm^−2^. As shown in Figure 4a, the addition of trace amounts of theaflavin enabled the Zn-Cu asymmetric cell to maintain stable operation for over 1000 charge–discharge cycles, with a coulombic efficiency consistently close to 100%. This performance markedly surpassed that of the control system using pure ZnSO_4_ electrolyte, which remained stable for only approximately 200 cycles before failure. Further details are provided in Figure 4b,c. In the electrolyte system containing theaflavin, the coulombic efficiencies recorded at the 1st, 300th, and 950th cycles were 94%, 99%, and 99%, respectively. In contrast, the system utilizing pure ZnSO_4_ exhibited lower coulombic efficiencies of 80%, 98%, and 85% at the 1st, 100th, and 216th cycles, respectively. The stabilizing effect of theaflavin on zinc-ion battery performance was further corroborated by the cycling behavior of Zn-Zn symmetric battery system (Figure 4d). Specifically, when the electrolyte was supplemented with theaflavin, the Zn-Zn symmetric battery system (1 mA·cm^−2^; 1 mAh·cm^−2^) demonstrated stable cycling performance exceeding 3000 h, with only a slight increase in the charge–discharge potential over time. Conversely, in the absence of theaflavin, the Zn-Zn symmetric battery system failed after approximately 270 h of operation.

Comparable results were observed under higher current density conditions. As illustrated in Figure S4, at a current density of 5 mA·cm^−2^, the system containing theaflavin electrolyte maintained stable operation for approximately 900 h. In contrast, the conventional ZnSO_4_ electrolyte system failed after only 165 h of cycling. Based on the performance at both 1 and 5 mA·cm^−2^, it was apparent that the theaflavin additive offered a notable enhancement in cycling stability. When compared with the reported stability efficiencies of other battery additives [38,39,40,48,49,50,51,52,53,54] (Figure 4f), the theaflavins investigated in this study demonstrated a relatively high level of effectiveness. The similar stability optimization action could also be observed from the rate performance of Zn-Zn symmetric battery system (unit: mA·cm^−2^; mAh·cm^−2^). As illustrated in Figure 4e, even after being subjected to stepwise increases in current density to 2, 4, and 8 mA·cm^−2^ (at 1 mAh·cm^−2^), the theaflavin-containing system maintained stable cycling performance for over 400 h. In contrast, the system with pure ZnSO_4_ electrolyte experienced rapid failure under the same conditions. Further insights were provided by XRD analysis (Figure 4g), which revealed the formation of substantial amounts of by-products, identified as ((Zn(OH)_2_)_3_(ZnSO_4_)·5H_2_O), in the pure ZnSO_4_ system after only 250 h of operation. These by-products were likely responsible for the premature degradation of the system. In comparison, the theaflavin-containing system showed only minimal by-product formation even after 2500 h of continuous cycling, further highlighting its superior stability.

**Figure 5:**
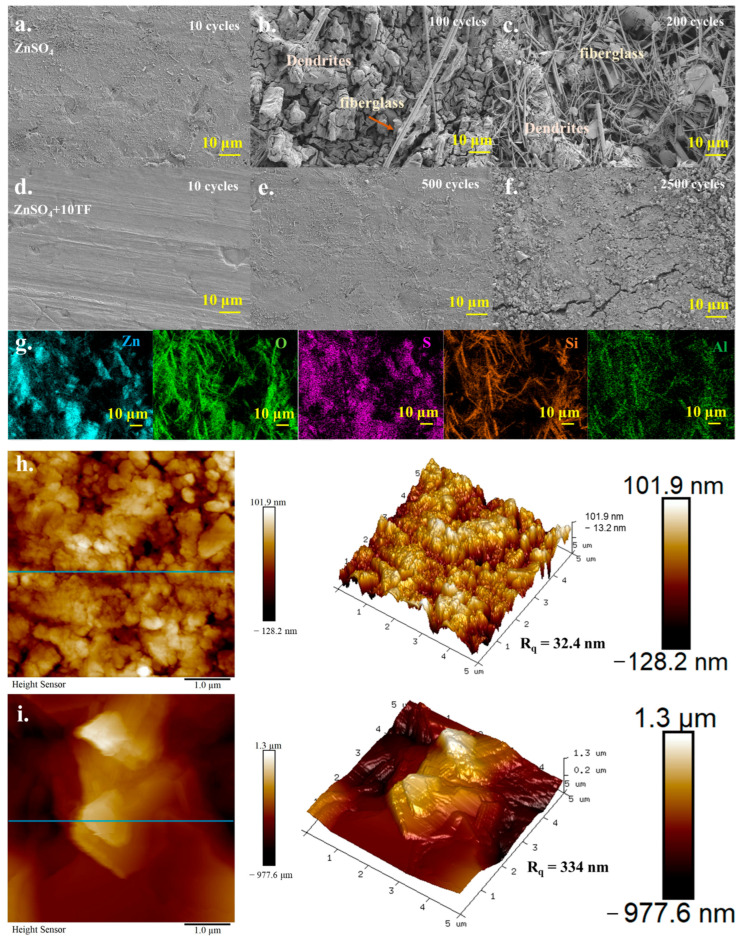
The SEM analysis results of Zn sheet after Zn-Zn symmetric battery system testing in ZnSO_4_ system ((**a**): after 10 cycles; (**b**): after 100 cycles and (**c**): after 200 cycles); The SEM analysis results of Zn sheet after Zn-Zn symmetric battery system testing in ZnSO_4_+10TF system ((**d**): after 10 cycles; (**e**): after 500 cycles and (**f**): after 2500 cycles); The SEM-EDX mapping analysis results of Figure 5c area (**g**); the AFM analysis results of Zn sheet after Zn-Zn symmetric battery system testing in ZnSO_4_ system (**i**) and ZnSO_4_+10TF system (**h**).

Additionally, comparable stability related observations were obtained from SEM-EDX analysis. As shown in Figure 5a–c, in the ZnSO_4_ electrolyte system, a significant accumulation of surface by-products was observed on the surface of zinc sheet after 100 cycles. Following 200 cycles, these deposits became more pronounced and were accompanied by fibrous structures. EDX elemental mapping (Figure 5g) revealed that the block-shaped by-products primarily consisted of zinc, sulfur, and oxygen, suggesting the formation of zinc dendrites composed of Zn(OH)_2_)_3_(ZnSO_4_)·5H_2_O. In contrast, the fibrous materials were mainly composed of silicon, aluminum, and oxygen, indicating the presence of fiberglass. The observed morphology revealed that the disordered growth of zinc dendrites had begun to penetrate the battery separator, potentially resulting in catastrophic battery failure. In contrast, in the ZnSO_4_+10TF system (Figure 5d–f), zinc dendrites were scarcely observable on the surface of the zinc sheet even after 500 cycles. Although dendrite formation was detected after 2500 cycles, their quantity remained significantly lower compared to that observed in the ZnSO_4_ system. To further investigate the surface characteristics of the zinc sheet post-cycling, atomic force microscopy (AFM) analysis was conducted. As illustrated in Figure 5h,i, the surface roughness of the zinc sheet cycled in the ZnSO_4_+10TF electrolyte ranged from −128.2 to 101.9 nm, with a root mean square roughness (Rq) of 32.4 nm, which was markedly lower than that observed for the sheet cycled in pure ZnSO_4_ electrolyte (0.9776 to 1.3 μm, Rq = 334 nm). Comparable results are presented in Figure S5. Analysis along the indicated lines (blue lines in Figure 5h,i) further proved that the vertical fluctuation amplitude on the zinc surface in the ZnSO_4_+10TF system was significantly reduced relative to the ZnSO_4_ system. These findings clearly implied that the incorporation of theaflavin effectively suppressed the formation of undesirable by-products and facilitated the development of a more stable electrochemical environment. Collectively, these results affirmed the positive role of theaflavin in enhancing the cycling stability and overall performance of zinc-ion batteries.

**Figure 6:**
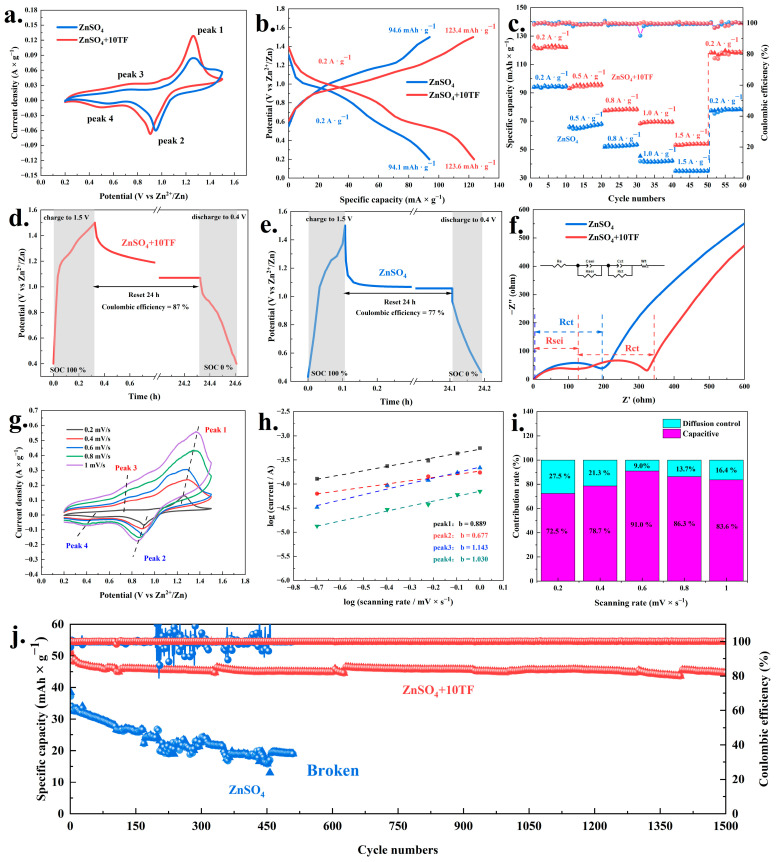
The electrochemical performance of zinc ion batteries based on V_2_O_5_ under ZnSO_4_ and ZnSO_4_+10TF system: CV (cyclic voltammetry) scanning curves (**a**); GCD (galvanostatic charge-discharge) curves ((**b**), 0.2 A·g^−1^); the rate performance of 0.2 to 1.5 A·g^−1^ (**c**); the self-discharge performance of ZnSO_4_+10TF system (**d**) and ZnSO_4_ system (**e**); The EIS (electrochemical impedance spectroscopy) curves and equivalent circuit model (Rct means charge transfer resistance and Rsei means solid electrolyte interphase resistance) (**f**); the CV scanning curves under different rate of ZnSO_4_+10TF system (**g**); log(i) vs. log(v) plots at main redox peak currents of ZnSO_4_+10TF system (**h**); the capacitive and diffusion contribution at different scan rates of ZnSO_4_+10TF system (**i**) and the Long cycle performance ((**j**), 2 A·g^−1^).

Based on the aforementioned studies, a full-cell investigation utilizing commercial V_2_O_5_ as the active material was carried out, with the corresponding results presented in Figure 6. As shown in Figure 6a, the CV curves of the battery within the voltage range of 0–1.5 V revealed two distinct pairs of redox peaks (peak1/peak2 and peak3/peak4). These peaks were commonly attributed to the redox transitions of V^3+^/V^4+^ and V^4+^/V^5+^, which were characteristic of vanadium-based systems in aqueous zinc-ion batteries. The intensities of these redox peaks presented a positive correlation with the battery’s specific capacity [27,44]. Notably, the introduction of theaflavin into the electrolyte significantly enhanced the intensity of both redox peaks, thereby contributing to an improved specific capacity. Subsequently, as shown in Figure 6b, when using pure ZnSO_4_ electrolyte (2 mol/L), the battery exhibited charge–discharge specific capacities of 94.6 and 94.1 mAh·g^−1^ at current density of 0.2 A·g^−1^, respectively. Upon the introduction of a trace amount of theaflavin (10 mmol/L) into the electrolyte, the specific capacities significantly increased to 123.4 and 123.6 mAh·g^−1^, respectively. These results clearly demonstrated that the incorporation of theaflavin effectively enhanced battery performance. A similar trend was observed in the rate-capability analysis (Figure 6c). In the pure ZnSO_4_ electrolyte system, the specific capacity declined sharply with increasing current density. At a high current density of 1.5 A·g^−1^, the specific capacity dropped to approximately 35 mAh·g^−1^. Furthermore, after reducing the current density back to 0.2 A·g^−1^, the recovered capacity was only about 78 mAh·g^−1^, indicating limited reversibility in the absence of theaflavin. In contrast, when the electrolyte contained both ZnSO_4_ and theaflavin, the battery exhibited significantly enhanced rate performance. Even at a high current density of 1.5 A·g^−1^, the specific capacity was maintained at approximately 53 mAh·g^−1^. Moreover, upon returning the current density to 0.2 A·g^−1^, the specific capacity rapidly recovered to around 120 mAh·g^−1^. These results indicated that the incorporation of trace amounts of theaflavin not only improved the specific capacity, but also markedly enhanced the long-term cycling stability of the battery. As shown in Figure 6j, when theaflavin was introduced into the electrolyte, the battery retained a stable specific capacity of approximately 45 mAh·g^−1^ after 1500 charge–discharge cycles at 2 A·g^−1^, while maintaining a coulombic efficiency close to 100%. In comparison, when using pure ZnSO_4_ electrolyte, the specific capacity of the battery declined rapidly with continuous charge–discharge cycling, and battery failure occurred after approximately 510 cycles. The enhanced long-term cycling stability observed with theaflavin addition was probably attributed to its previously discussed roles in suppressing zinc dendrite formation and mitigating the HER. Moreover, the introduction of trace amounts of theaflavin into the electrolyte significantly improved the battery’s self-discharge performance, as evidenced by the results shown in Figure 6d,e. Specifically, in the presence of theaflavin, the coulombic efficiency remained as high as 87% after the battery was fully charged and left to stand for 24 h. In contrast, under identical conditions with only ZnSO_4_ electrolyte, the coulombic efficiency decreased to 77%.

Furthermore, the results of electrochemical impedance spectroscopy are depicted in Figure 6f and Figure S6. As observed, the solution resistance (Rs) for both electrolyte systems was approximately 2.8 Ω. In the case of the pure ZnSO_4_ electrolyte, the Nyquist plot exhibited a single semicircle, corresponding solely to charge transfer resistance (Rct), which was measured to be approximately 248 Ω, in addition to the inherent internal resistance. In contrast, the EIS spectrum of the theaflavin-containing electrolyte displayed two distinct semicircles. This implied the presence of both charge transfer resistance (Rct: about 205 Ω) and an additional resistance component, Rsei (210 Ω), attributed to the formation of a solid electrolyte interphase layer on the surface of the zinc anode. The formation of this organic interfacial film was considered highly advantageous, as it played a critical role in suppressing zinc dendrite growth [55,56]. Subsequently, the CV curves of the battery system at various scan rates were presented in Figure 6g and Figure S7. It was clear that the current response increased with rising scan rate, indicating enhanced electrochemical activity. According to the Bruce–Dunn kinetic process analysis method [57], the peak current of the oxidation-reduction peak in the CV curves displayed a power-law relationship with the scan rate, as described by Equations (1) and (2) [58].

(1) $$I=av^{b}$$

(2) log(I)=blog(v)+log(a)

Here, *ν* denotes the scan rate, *I* represents the peak current, and a is an adjustable parameter. Equation (2) is a logarithmic transformation of Equation (1).

Based on the calculated results derived from the above equations (Figure 6h and Figure S8), the b values corresponding to the four redox peaks in the theaflavin-containing electrolyte system were determined to be 0.889, 0.677, 1.143, and 1.030, respectively. These values were notably higher than those observed for the pure ZnSO_4_ electrolyte system, which were 0.687, 0.496, 0.638, and 0.784, respectively. Theoretically, a b value close to 0.5 indicated a diffusion controlled process, whereas a b value approaching 1 suggested a surface (capacitive)-controlled behavior [59]. Evidently, both electrolyte systems presented mixed control mechanisms, as their average b values fell between 0.5 and 1. However, the comparatively higher b values observed in the theaflavin containing system implied a greater contribution from surface-controlled processes. To further quantify the respective contributions of capacitive and diffusion-controlled mechanisms, Equations (3) and (4) were employed for analysis [60,61].

(3) $$i=k_{1}v+k_{2}v^{0.5}$$

(4) $$i/v^{0.5}=k_{1}v^{0.5}+k_{2}$$

where *ν* represents the scanning rate, *i* represents the peak current, and *k*_1_ and *k*_2_ are two coefficients.

According to the fitting calculations, the diffusion-controlled contributions in the theaflavin containing electrolyte system were determined to be 27.5%, 21.3%, 9.0%, 13.7%, and 16.4% at various scan rates (Figure 6i), which were markedly lower than those observed in the pure ZnSO_4_ system, 71.2%, 61.4%, 57.6%, 44.6%, and 62%, respectively (Figure S9). These results further confirmed that the presence of theaflavins promoted a transition toward surface-dominated electrochemical behavior. This behavior could be attributed to the enhanced zinc ion diffusion rate facilitated by the presence of theaflavins, which promoted the de-solvation of zinc ions and thereby accelerated ion transport. Overall, the comprehensive electrochemical performance of the full cell system demonstrated that the incorporation of theaflavins significantly promoted the redox activity of vanadium-based active materials and improved zinc ion diffusion kinetics. As a result, notable enhancements in specific capacity, rate capability, long-term cycling stability, and self-discharge performance were achieved. These findings clearly indicated that the addition of theaflavins was highly beneficial for enhancing the overall performance of vanadium-based zinc-ion batteries.

**Figure 7:**
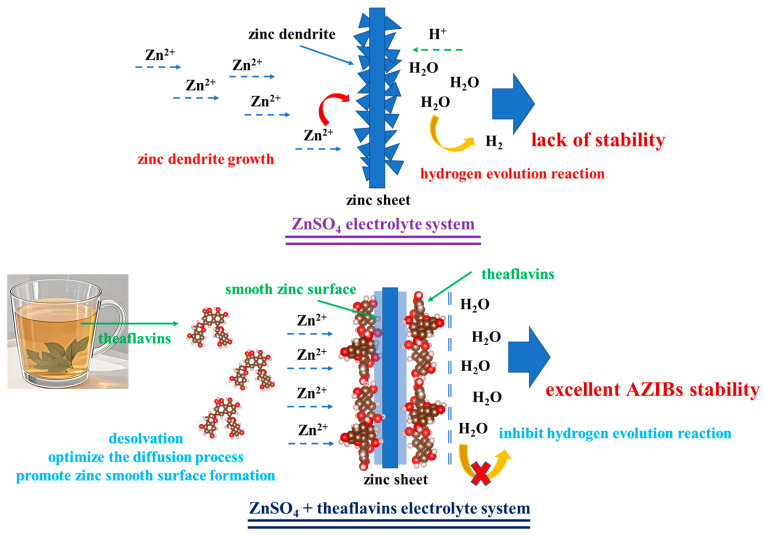
The mechanism diagram of theaflavins promoting the stability of AZIBs (aqueous zinc-ion batteries).

Based on the above findings, the mechanism by which theaflavins enhanced the stability of aqueous zinc ion batteries is illustrated in Figure 7. As shown, in the pure ZnSO_4_ electrolyte system, the irregular diffusion of zinc ions and the absence of protective surface layers resulted in the uncontrolled growth of zinc dendrites on the zinc anode during cycling. These dendrites eventually penetrated the separator, leading to internal short-circuits. Additionally, electrochemical hydrogen evolution reactions inevitably occurred on the zinc surface, contributing to the decomposition of the ZnSO_4_ electrolyte and further compromising battery performance. Ultimately, these two detrimental effects, uncontrolled dendrite growth and hydrogen evolution, led to the failure of the battery system. In contrast, when theaflavins were introduced into the electrolyte, their strong de-solvation capability facilitated the more uniform and stable diffusion of zinc ions, effectively suppressing dendrite formation. Additionally, the preferential adsorption of theaflavin molecules onto the zinc anode surface formed a protective layer that minimized direct contact between water molecules and the zinc surface. This not only enhanced interfacial stability but also significantly mitigated electrochemical hydrogen evolution. In summary, the incorporation of theaflavins effectively inhibited both zinc dendrite growth and hydrogen evolution reactions, thereby contributing to the superior cycling stability of the zinc-ion battery system investigated in this study.

## 3. Materials and Methods

The reagents used in this study included theaflavins (Aladdin, purity > 98%, the remaining impurities are unknown, but they are also polyphenol extracts derived from tea leaves), zinc sulfate heptahydrate (ZnSO_4_·7H_2_O) (National Pharmaceutical Reagents, analytical grade), polyvinylidene fluoride (PVDF) (Aladdin, analytical grade), carbon black (National Pharmaceutical Reagents, analytical grade), N-methyl-2-pyrrolidone (NMP) (Aladdin, analytical grade, purity > 99%), and vanadium pentoxide (V_2_O_5_) (Aladdin, analytical grade, purity > 99%). The zinc and copper sheets used in the experiments were 0.1 mm thick. Prior to use, they were thoroughly cleaned with deionized water and ethanol, polished to achieve a smooth surface on both sides, and cut into 13 mm circular disks using a precision slicer.

The preparation process of the positive electrode sheet for the full battery was as follows: V_2_O_5_, carbon black, and PVDF were mixed in a 7:2:1 mass ratio and ground uniformly in an agate mortar. The resulting mixture was then transferred to a glass bottle and combined with approximately 0.5 mL of NMP, followed by magnetic stirring for 24 h to ensure homogeneity. Subsequently, the uniformly dispersed slurry was coated onto the surface of titanium foil (0.02 mm thickness) and dried in a vacuum oven for 12 h to obtain the positive electrode sheet. Finally, the dried electrode sheet was cut into 13 mm diameter circular pieces using a precision slicer, with each piece containing an active material loading of approximately 1 mg for further use.

The electrochemical experiments conducted encompassed Tafel analysis, chronoamperometry, hydrogen evolution reaction, and electrochemical impedance spectroscopy. The concentration of theaflavins in the electrolyte was determined to be 10 mmol/L based on preliminary experimental results (Figure S10 and related discussion). The Tafel analysis was performed using a three-electrode system, comprising a zinc sheet as the working electrode, a platinum wire as the counter-electrode, and an Ag/AgCl electrode as the reference electrode. The electrolytes employed for this analysis were 2 mol/L ZnSO_4_ solution and a mixture of 2 mol/L ZnSO_4_+10 mmol/L theaflavins. The experimental procedure involved initial measurement of the open-circuit potential of the system, followed by Tafel testing conducted within a potential range of ±0.3 V relative to the open circuit potential, at a scan rate of 5 mV/s. The corrosion current density was subsequently determined using calculation software integrated with the electrochemical workstation. For the HER, the same three-electrode configuration was utilized, with electrolytes consisting of 1 mol/L Na_2_SO_4_ and a mixture of 1 mol/L Na_2_SO_4_+10 mmol/L theaflavins. The testing potential range for HER was set from −0.9 V to −1.6 V versus the Ag/AgCl reference electrode. In all electrochemical experiments, the zinc sheet working electrode (length: 1 cm, width: 1 cm, thickness: 0.1 mm) was prepared by a special hydraulic slicer and both sides of the zinc sheet working electrode were immersed in the corresponding electrolyte. The chronoamperometry measurements were conducted using a two-electrode system, where a zinc sheet served as the working electrode, and an identical zinc sheet functioned as both the reference and counter-electrode. The electrolyte solutions used for testing included a 2 mol/L ZnSO_4_ solution and a 2 mol/L ZnSO_4_+10 mmol/L theaflavin mixture. In order to study the diffusion behavior of zinc ions in different electrolytes, referring to the relevant literature [30,34], the applied potential was set at −0.15 V versus the reference electrode, with a measurement duration of 300 s.

Furthermore, battery performance testing was conducted using assembled CR2032 button cells. Before assembly, the purchased zinc foil (thickness: 0.1 mm) was cut into 13 mm circular pieces using a dedicated hydraulic slicer. The assembly sequence of the button cell was as follows: positive shell, positive electrode, separator (GF/A), zinc plate (serving as the negative electrode), gasket, spring plate, and negative shell. The electrolyte volume used for each cell was 100 μL, consisting of either a 2 mol/L ZnSO_4_ solution or a 2 mol/L ZnSO_4_ + 10 mmol/L theaflavin mixture. Both sides of the electrode were wetted by electrolyte. The assembly pressure applied to the battery was 50 kg/cm^2^. For coulombic efficiency testing, a copper sheet was used as the positive electrode. The discharge current density was set to 1 mA/cm^2^, with a discharge duration of 1 h. The charging current was also 1 mA/cm^2^, with a cut-off voltage of 1 V. The stability of zinc dissolution and deposition was evaluated using a Zn-Zn symmetric battery system, in which a zinc sheet served as the positive electrode. The testing conditions included: (a) charge and discharge current densities of 1 mA/cm^2^ with charge and discharge capacities of 1 mAh/cm^2^; (b) charge and discharge current densities of 55 mA/cm^2^ with charge and discharge capacities of 1 mAh/cm^2^; and (c) stepwise charging and discharging currents of 1, 2, 4, and 8 mA/cm^2^, followed by a return to 1 mA/cm^2^, with charge and discharge capacities of 1 mAh/cm^2^. The schematic diagram of CR2032 button battery system assembly is described in Figure S11. All tests were conducted until battery failure occurred.

The influence of theaflavins on battery capacity was evaluated using a full-cell system, in which the positive electrode consisted of a titanium sheet loaded with commercial V_2_O_5_ (prepared as previously described). The electrochemical characterization methods included cyclic voltammetry (CV), electrochemical impedance spectroscopy (EIS), galvanostatic charge–discharge (GCD), rate performance analysis, and long-cycle stability testing. CV scans were conducted within a voltage range of 0.2 to 1.5 V at scan rates of 0.2, 0.4, 0.6, 0.8, and 1 mV·s^−1^. EIS measurements were performed with an amplitude of 0.005 V over a frequency range of 0.01 to 100,000 Hz. GCD tests, including rate performance evaluations, were carried out at current densities of 0.2, 0.5, 0.8, 1, and 1.5 A·g^−1^ within a voltage range of 0.2 to 1.5 V. Long-cycle stability was assessed over 1500 GCD cycles at a current density of 2 A·g^−1^. Finally, self-discharge behavior was evaluated by charging the battery to 1.5 V, allowing it to rest for 24 h, and then discharging it to 0.2 V.

To conduct a comprehensive analysis of the molecular properties of theaflavins and their adsorption behavior on a zinc sheet, classical molecular dynamics (MD) simulations and density functional theory (DFT) calculations were employed. Initially, classical MD simulations were performed using the GROMACS software 2024.3 [62]. The force field parameters were derived from the Amber force field, and the SPC/E water model was utilized for H_2_O molecules. The simulation box had dimensions of 43 × 43 × 43 Å^3^, with periodic boundary conditions applied in all three directions. The simulation system comprised 2120 H_2_O, 80 Zn^2+^ ions, 80 SO_4_^2−^ ions, and 4 theaflavin molecules. Electrostatic interactions were calculated using the Particle Mesh Ewald (PME) method, with a cutoff distance of 1.0 nm for both electrostatic and non-electrostatic interactions in real space. The integration time step was set to 1 fs, and the total simulation time was 450 ps. The simulations were conducted at a temperature of 298 K and a pressure of 1.01 × 10^5^ Pa. Temperature and pressure coupling were implemented using the V-rescale and Berendsen methods, respectively.

Density Functional Theory (DFT), a first-principles computational approach based on electron density, was employed using the Vienna Ab initio Simulation Package (VASP) [63]. This method assumed that the ground-state properties of electrons were entirely determined by their electron density. In practical computations, approximate exchange-correlation functionals, such as the generalized gradient approximation (GGA) [64], were utilized to account for electron–electron interactions. The computational process commenced with the construction and optimization of theoretical molecular structures (unit cells) for theaflavins and zinc. Subsequently, calculations were performed to determine the molecular electrostatic potential, optimal adsorption configuration, and adsorption energy. The exchange-correlation potential was evaluated using the Perdew–Burke–Ernzerhof (PBE) functional [65,66] within the generalized gradient approximation (GGA) framework. A K-point grid of 4 × 4 × 4 and a cut-off energy of 450 eV were employed throughout the calculations. Figure S12a presents the theoretical structure of the theaflavin molecule, which belonged to the space group P1, with lattice constants defined as a = b = c = 16.738 Å, along with alpha = beta = gamma = 90.0°. The molecular structure consisted of 12 oxygen atoms, 29 carbon atoms, and 24 hydrogen atoms. The electrostatic potential of the theaflavin molecule was calculated using a combination of VASP (version 5.4.4), Multiwfn (version 3.7) [67,68], and Visual Molecular Dynamics (VMD) software (version 1.9.3) [69]. Figure S12b illustrates the original theoretical structure of zinc, with a space group identified as P 63/mmc and lattice constants delineated as follows: a = 2.78953 Å, b = 2.78953 Å, c = 4.33339 Å, along with alpha = 90.0°, beta = 90.0°, and gamma = 120.0°. The original molecule cell contained only 2 zinc atoms and then 8 × 8 × 1 supercell was built. Based on the supercell, the 002, 100 and 101 surfaces of zinc sheets were constructed (with a vacuum layer of 40 Å and 4 zinc atomic layers retained) for adsorption calculations (Figure S12c). The optimal configurations of theaflavins and water molecules adsorbed on the surfaces of zinc sheets (002, 100 and 101) after theoretical calculations are shown in Figures S13–S18. The valence electron structures of each atom were as follows: C—s2p2; O—s2p4; H—s1; and Zn—d10p2.

## 4. Conclusions

A low-cost and efficient organic polyphenol, theaflavins, derived from tea, was introduced into a typical ZnSO_4_ electrolyte as an additive, effectively suppressing the growth of zinc dendrites and mitigating the electrochemical hydrogen evolution reaction, thereby enhancing the interfacial stability in AZIBs. The theaflavin molecule, with its multiple hydroxyl groups, interacted strongly with Zn^2+^ and H_2_O, disrupting their solvation structure. This increased the availability of free Zn^2+^ and H_2_O molecules in the electrolyte, enhancing Zn^2+^ ion diffusion and significantly influencing electrolyte solvation effects. Considering the results from MD and DFT, NMR, and FT-IR, theaflavins preferentially adsorbed onto the zinc anode, facilitating more efficient electron transfer during zinc ion stripping and deposition. This was attributed to their stronger affinity with the zinc surface compared to water molecules. Moreover, the Zn/Cu asymmetric system achieved high coulombic efficiencies exceeding 95%. The Zn/Zn symmetric system exhibited remarkable cycling stability, operating for approximately 3000 h and 900 h at current densities of 1 and 5 mA/cm^2^, respectively. In full-cell configurations with V_2_O_5_ as the cathode material, the addition of theaflavins significantly enhanced specific capacity, rate performance, cycling stability, and self-discharge behavior. This highlighted their broad applicability and performance-enhancing effects in AZIBs.

## Data Availability

The original contributions presented in this study are included in the article/Supplementary Material. Further inquiries can be directed to the corresponding authors.

## Supplementary Materials

The following supporting information can be downloaded at: https://www.mdpi.com/article/10.3390/ijms26199399/s1.
