# Supplementary material for: Theaflavins as Electrolyte Additives for Inhibiting Zinc Dendrites and Hydrogen Evolution in Aqueous Zinc-Ion Batteries
Source: Int J Mol Sci. 2025 Sep 26;26(19):9399. doi: 10.3390/ijms26199399 (PMC12525372; doi:10.3390/ijms26199399)
Supplement: Supplementary file 1 [file ijms-26-09399-s001.zip › ijms-3838531-supplementary.pdf]

International Journal of
Molecular Sciences

MDPI

Theaflavins as electrolyte additives for inhibiting zinc dendrites and hydrogen evolution in aqueous zinc-ion batteries

Xiao Zhang[1,2], Ting Cheng[1,3], Chen Chen[3] *, Fuqiang Liu[2,4], Fei Wu[3], Li Song[1,2], Baoxuan Hou[3], Yuan Tian[3], Xin Zhao[3], Safi Ullah[3] and Rui Li[3]

[1] School of Environmental Ecology, The City Vocational College of Jiangsu; Jiangsu Engineering and Technology Centre for Ecological and Environmental Protection in Urban and Rural Water Environment Management and Low Carbon Development, Nanjing, 210017, P.R. China;
[2] State Key Laboratory of Pollution Control and Resource Reuse, School of Environment, Nanjing University, Nanjing, 210023, P. R. China;
[3] School of Environmental and Chemical Engineering, Jiangsu University of Science and Technology, Zhenjiang, 212100, P.R. China;
[4] Key Laboratory of Agro-Forestry Environmental Processes and Ecological Regulation of Hainan Province, School of Environmental Science and Engineering, Hainan University, Haikou, 570228, P. R. China.

* Chen Chen (ORCID: 0000-0003-3714-7076); Email: chenc@just.edu.cn.
Tel.: +86-0511-85639001; Fax: +86-0511-85639001

S1. Experiments and methods

S1.1 Materials Characterization

A range of advanced analytical techniques were employed to comprehensively investigate the properties of the battery materials. The mineral phase composition of the materials was determined by X-ray diffraction (XRD) analysis. The measurements were carried out on a Beijing Puxi XD-6 polycrystalline X-ray diffractometer, utilizing Cu-Kα radiation with a wavelength of 1.54056 Å. The morphological features and microelement composition of the materials were examined using scanning electron microscopy (SEM). The instruments used included a HITACHI Regulus 8100 SEM equipped with a Super-X EDS detector. The molecular structure and interactions within the electrolyte were analyzed by determining the hydrogen nuclear magnetic resonance (^{1}H-NMR) spectra using the Bruker AVANCE NEO 400M NMR spectrometer. The chemical composition and functional group analysis of organic compounds in battery materials were conducted using Fourier Transform Infrared Spectroscopy (FT-IR) with a Thermo Fisher Scientific Nicolet iS20 instrument. The surface morphology and microstructure analysis of electrode materials were performed using the Bruker Dimension Icon Atomic Force Microscope (AFM).

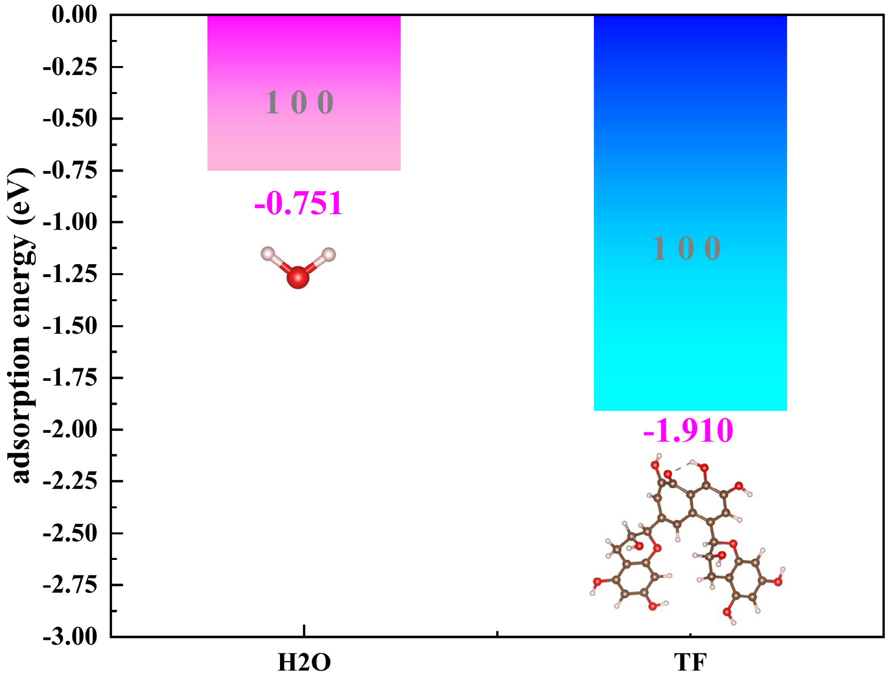

Figure S1. The theoretical adsorption energy DFT calculation results of H_2O and theaflavins molecules on the 100 crystal surface of zinc sheet.

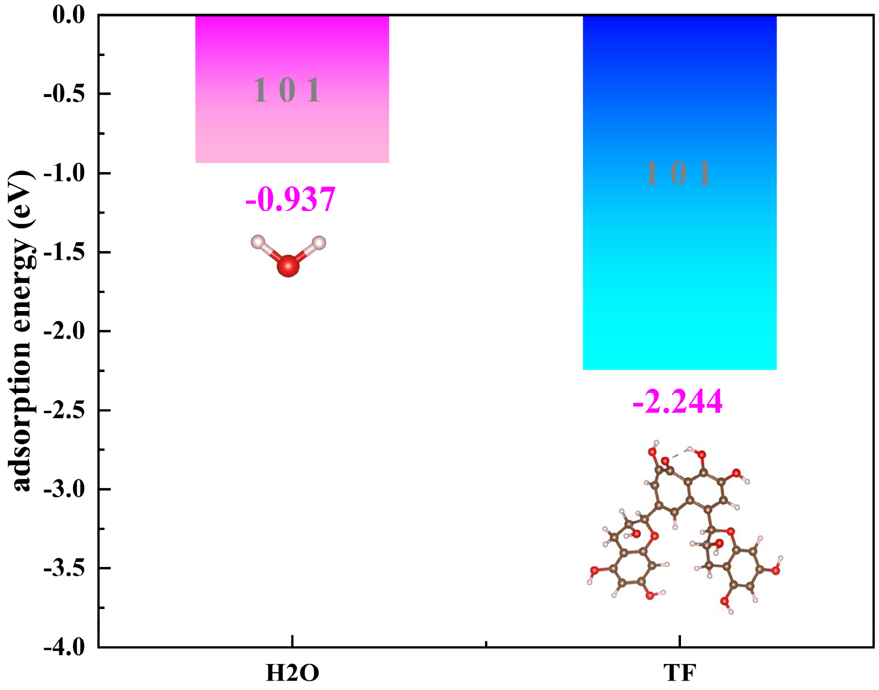

Figure S2. The theoretical adsorption energy DFT calculation results of H_2O and theaflavins molecules on the 101 crystal surface of zinc sheet.

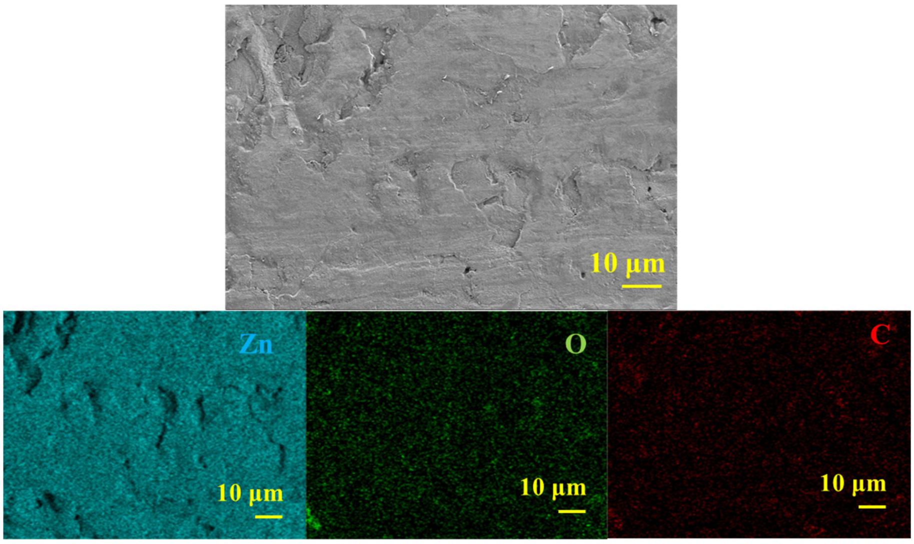

Figure S3. The EDX mapping analysis results of zinc sheet soaked in $ZnSO_4$+10TF solution.

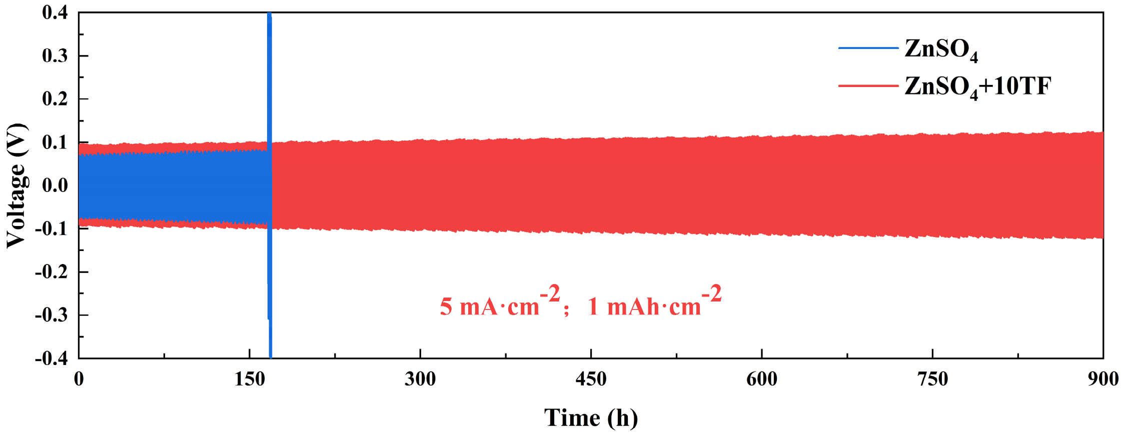

Figure S4. The long Zn-Zn symmetric battery system testing results (5 mA·cm^{-2} and 1mAh·cm^{-2}).

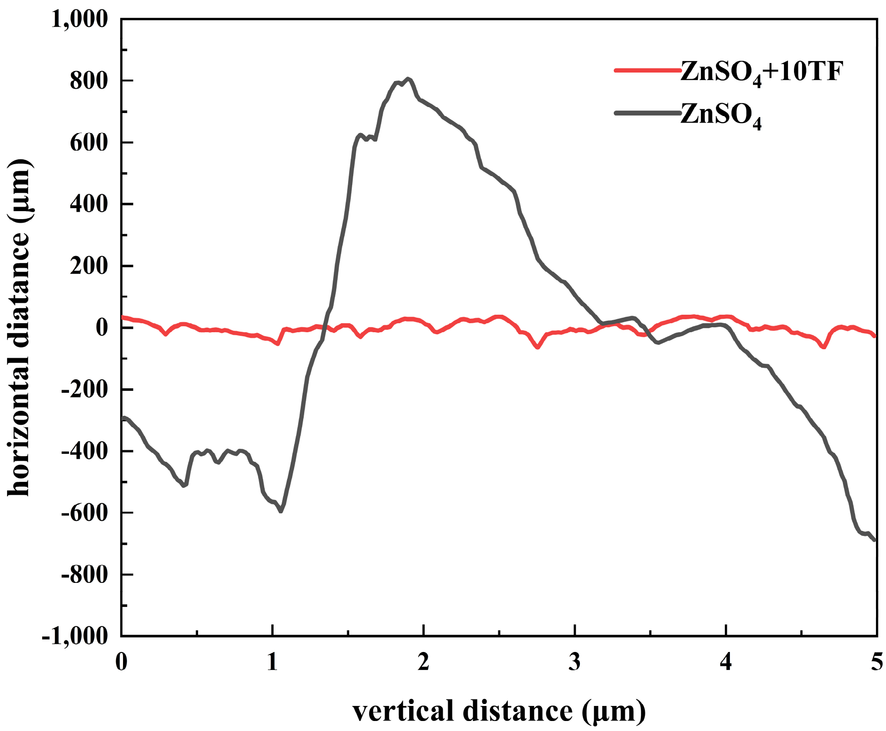

Figure S5. The test results of roughness along the analysis line in Fig.7h and 7j.

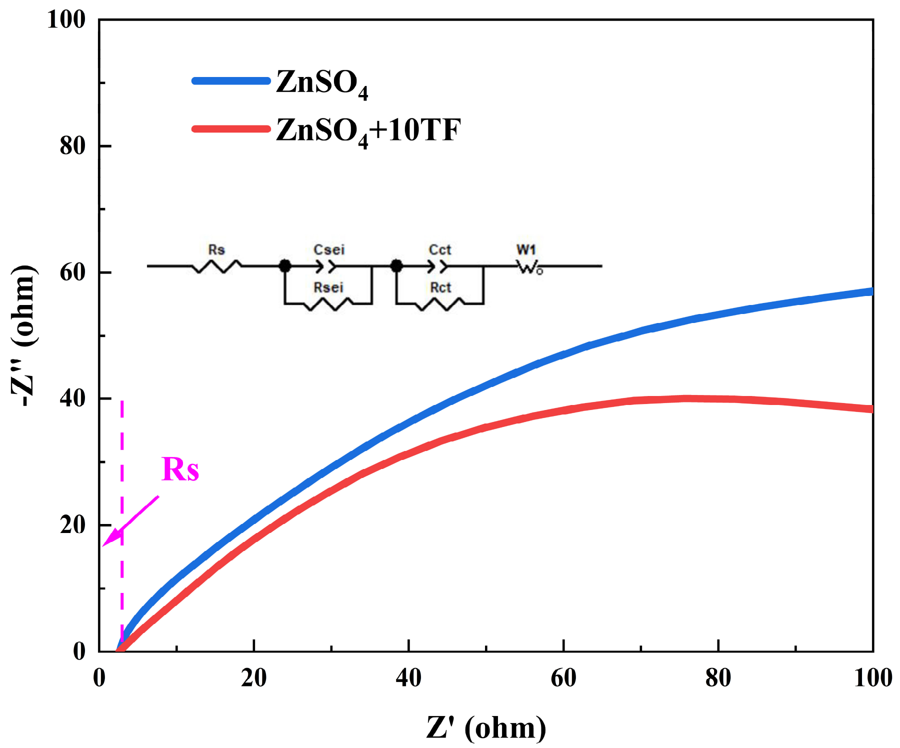

Figure S6. The EIS curves detail and equivalent circuit model (insert) of full battery system.

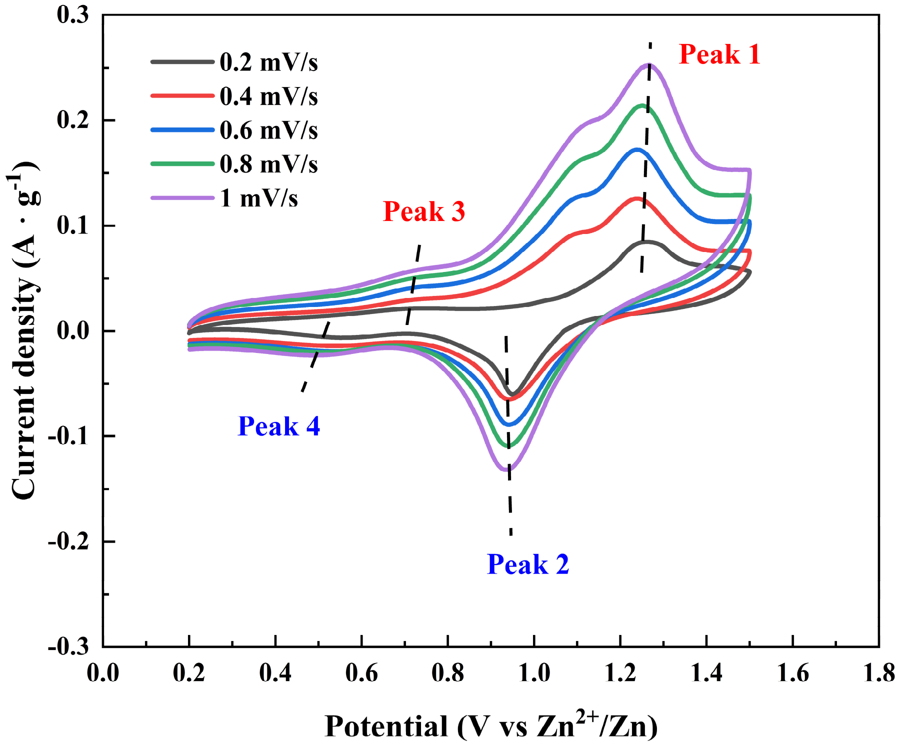

Figure S7. The CV scanning curves under different rate under $ZnSO_4$ system of full battery system.

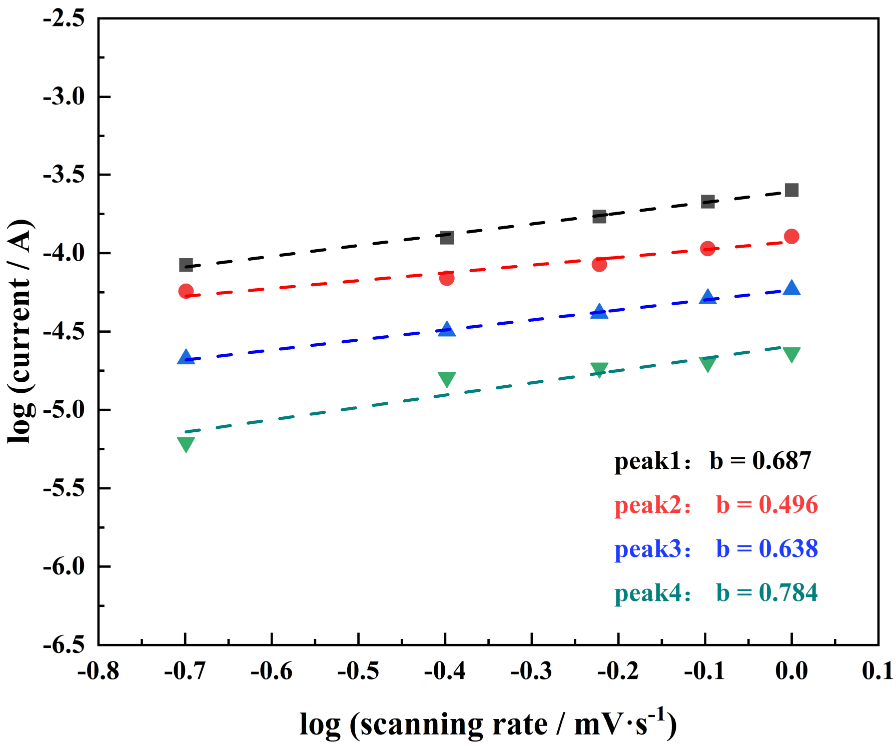

Figure S8. The log(i) vs log(v) plots at main redox peak currents under $ZnSO_4$ system of full battery system.

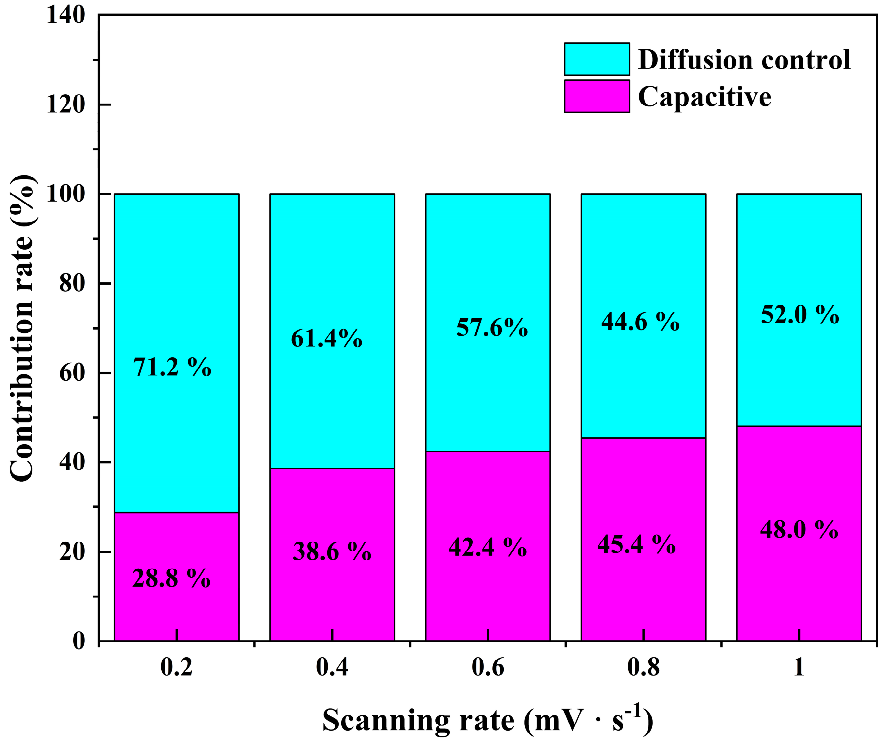

Figure S9. The capacitive and diffusion contribution at different scan rates under $ZnSO_4$ system of full battery system.

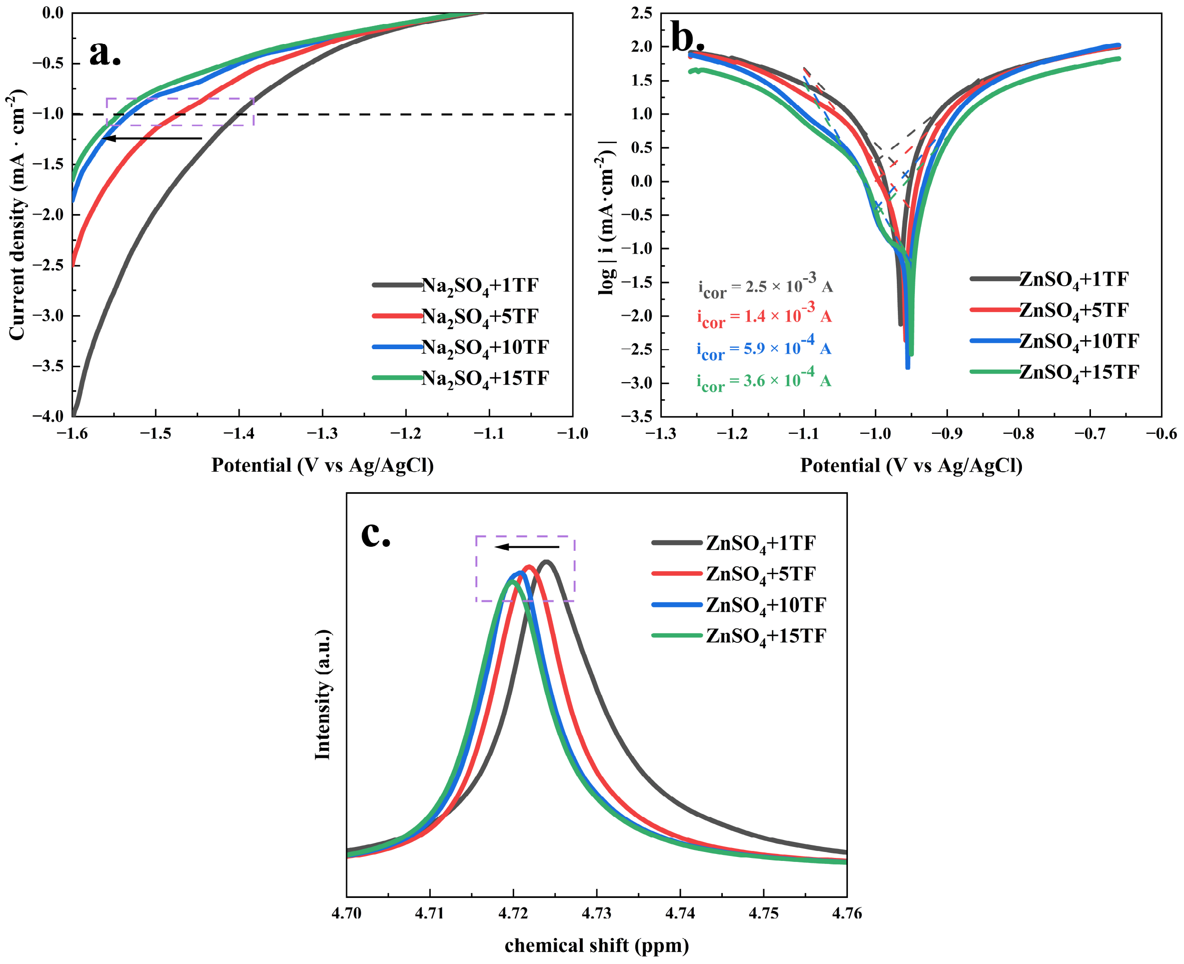

Figure S10. The preliminary study on the influence of theaflavins concentration on HER reaction (a), Tafel corrosion current (b), and ^2H (D) NMR analysis results (c) (the number before TF in the legend represents the concentration of theaflavins, in units of mmol/L).

Fig.S10 showed the initial research findings on the concentration of theaflavins. As the concentration of theaflavins increased from 1 mmol/L to 15 mmol/L, the overpotential of HER reaction increased (Fig.S10a), the Tafel corrosion current decreased (Fig.S10b), and the ^2H peak gradually shifted to the left (Fig.S10c). These changes collectively contributed to the enhanced stability of zinc ion batteries. Meanwhile, as the concentration of theaflavins increased, the marginal effect caused by the increase of concentration gradually weakened. Especially after the concentration increased to 10 mmol/L, the optimization amplitude of overpotential, Tafel corrosion current, and ^2H peak gradually weakened. Therefore, considering the optimization effect and their widespread application in the later stage, 10 mmol/L was chosen as the concentration of theaflavins added in this study.

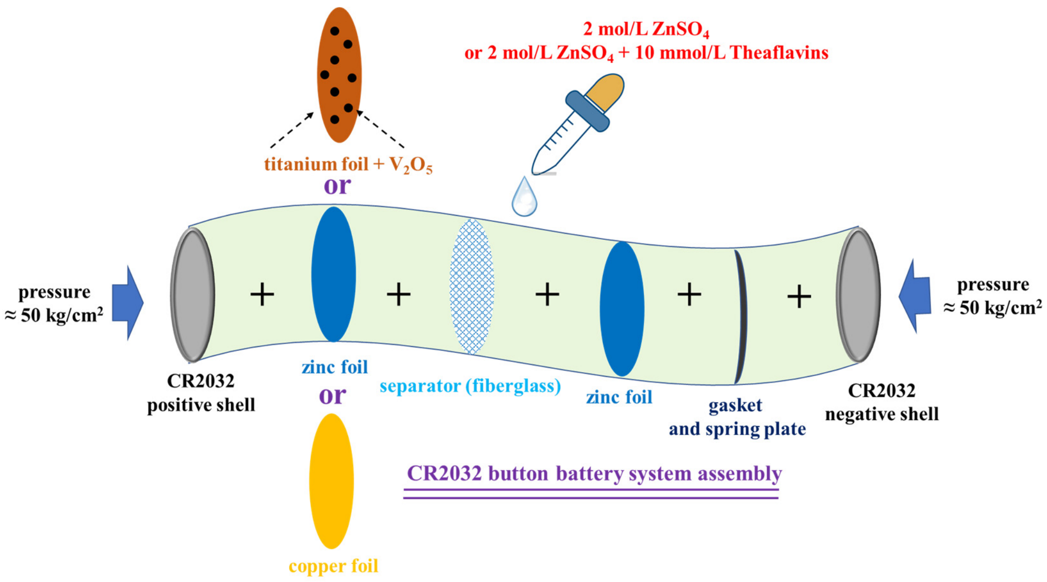

Figure S11. The schematic diagram of CR2032 button battery system assembly.

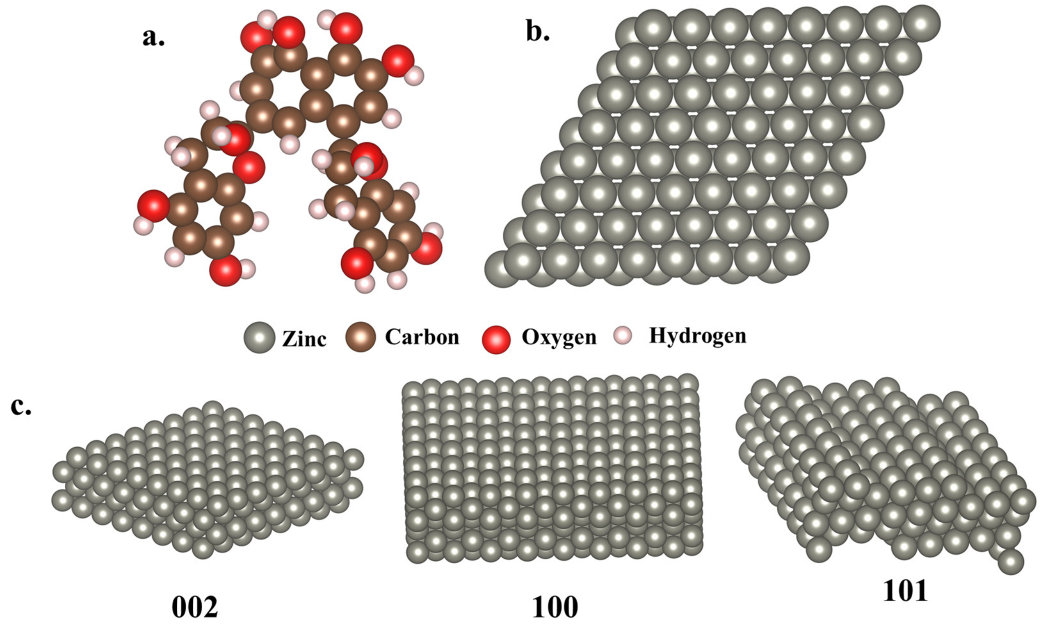

Figure S12. The theoretical model of theaflavins (a) and zinc sheet (b, 8 × 8 × 1 supercell); theoretical catalytic crystal planes of zinc sheet (c).

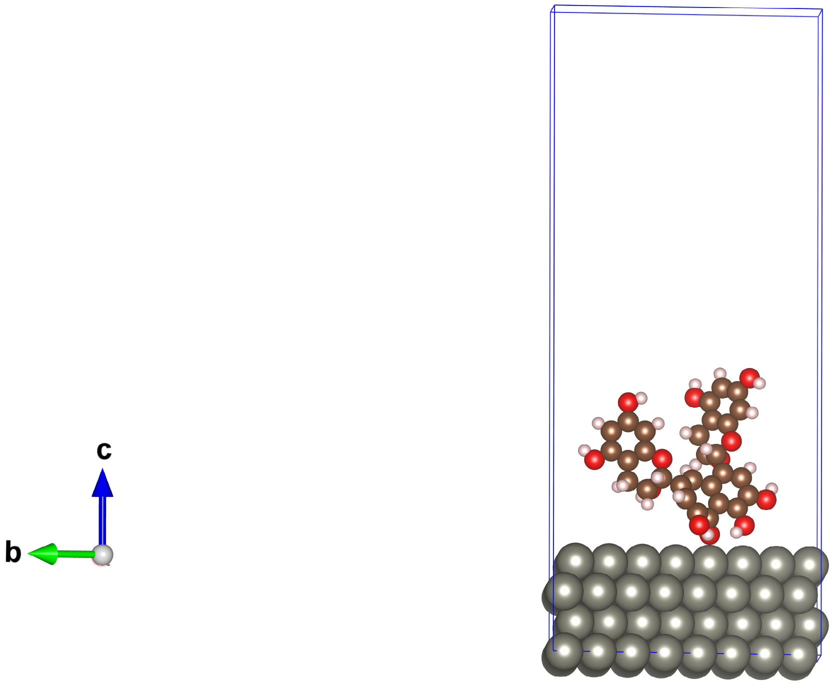

Figure S13. The optimal configurations of theaflavins molecules adsorbed on the surfaces of zinc sheets (002) after theoretical calculations.

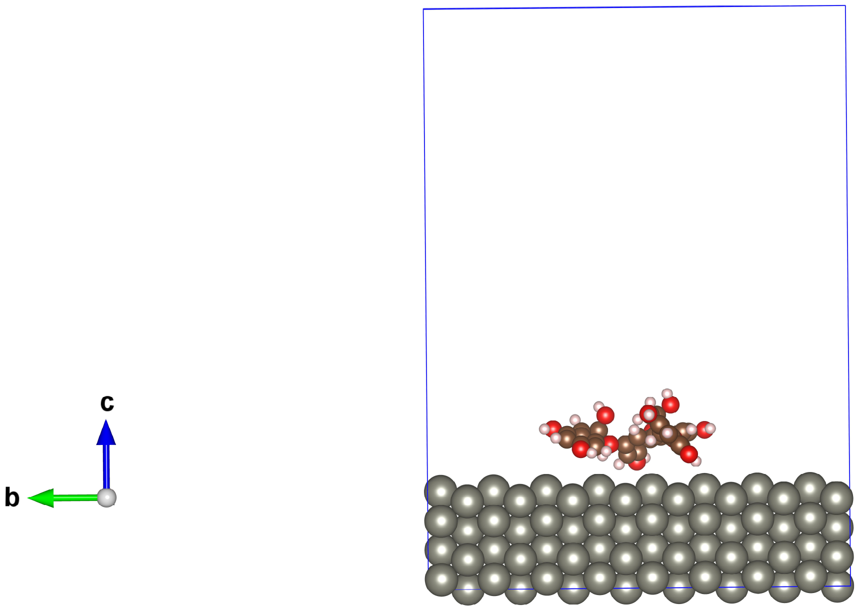

Figure S14. The optimal configurations of theaflavins molecules adsorbed on the surfaces of zinc sheets (100) after theoretical calculations.

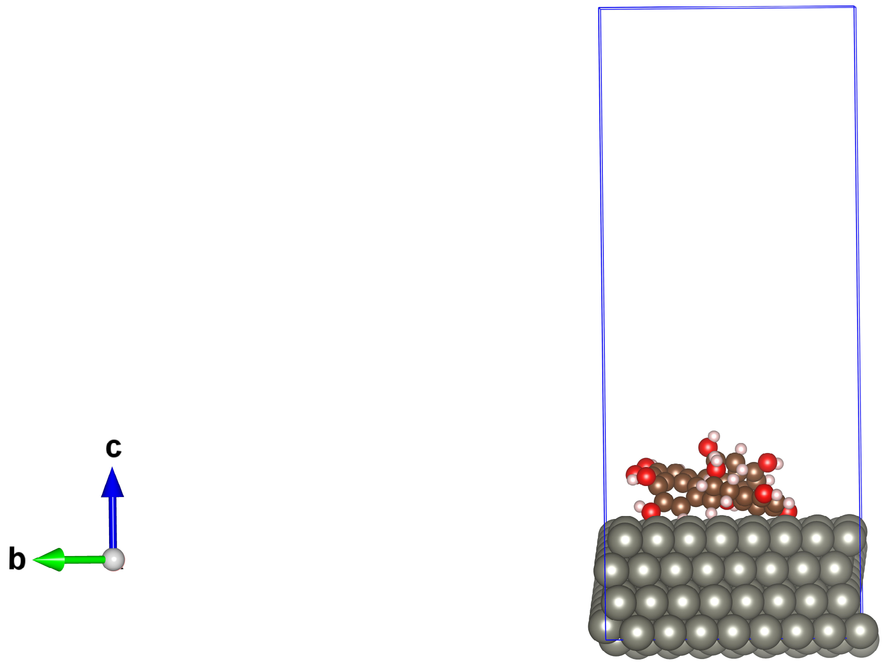

Figure S15. The optimal configurations of theaflavins molecules adsorbed on the surfaces of zinc sheets (101) after theoretical calculations.

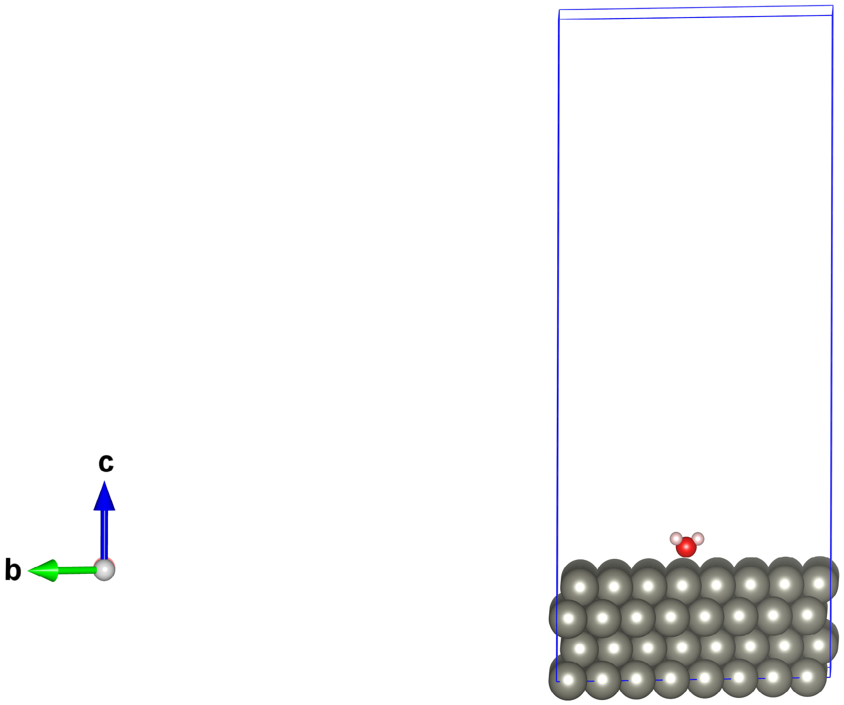

Figure S16. The optimal configurations of H_2O molecules adsorbed on the surfaces of zinc sheets (002) after theoretical calculations.

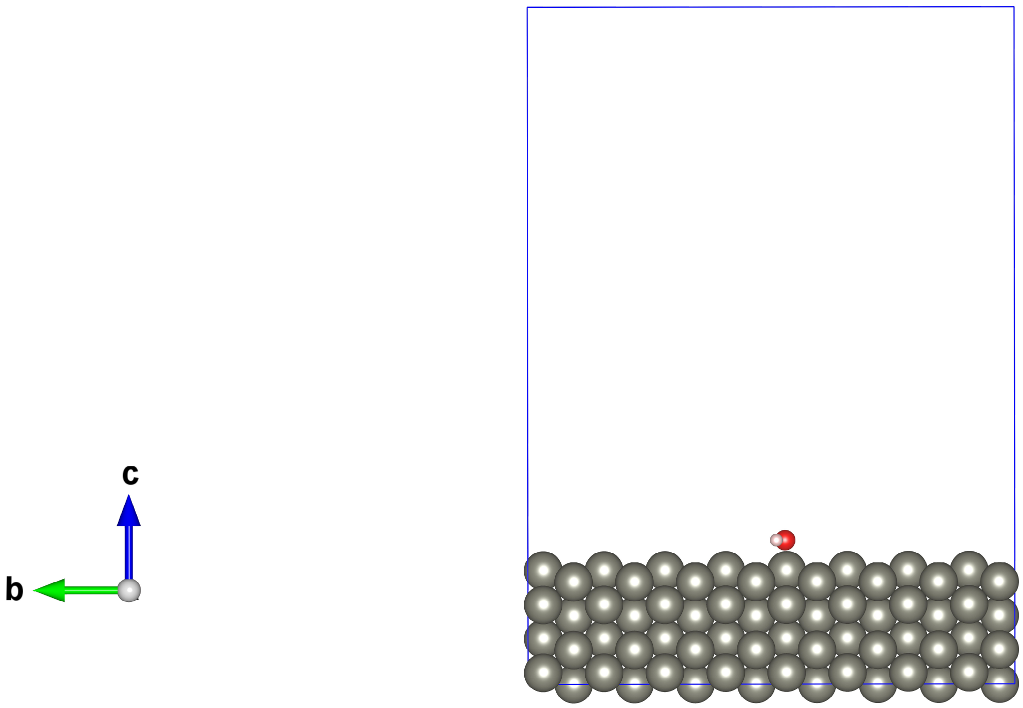

Figure S17. The optimal configurations of H_2O molecules adsorbed on the surfaces of zinc sheets (100) after theoretical calculations.

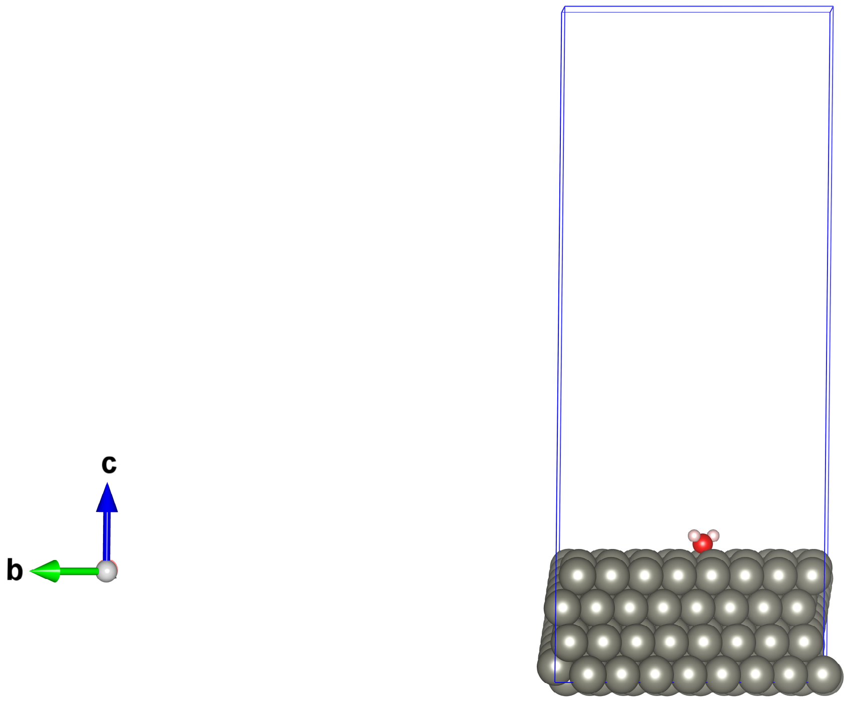

Figure S18. The optimal configurations of H_2O molecules adsorbed on the surfaces of zinc sheets (101) after theoretical calculations.

Disclaimer/Publisher's Note: The statements, opinions and data contained in all publications are solely those of the individual author(s) and contributor(s) and not of MDPI and/or the editor(s). MDPI and/or the editor(s) disclaim responsibility for any injury to people or property resulting from any ideas, methods, instructions or products referred to in the content.
